# Elimination of Epidemic Meningitis in the African Region: Progress and Challenges: 2010-2016

**Published:** 2018-07-02

**Authors:** Amadou Fall, André Fouda Bita, Clement Lingani, Mamoudou Djingarey, Carole Tevi-Benissan, Marie-Pierre Preziosi, Olivier Ronveaux, R. Mihigo, J. Okeibunor, Bartholomew Dicky Akanmori

**Affiliations:** 1 IST West Africa, WHO Regional Office for Africa, Ougadougou, Burkina Faso; 2 IVD/FRH, WHO Regional Office for Africa, Brazzaville, Congo; 3 IVR/IVB WHO HQ, Geneva, Switzerland; 4 World Health Organization. Department: Control of Epidemic Diseases. City: Geneva; 5 Polio Eradication Programme, WHO Regional Office for Africa, Brazzaville, Congo

**Keywords:** Epidemic Meningitis, Elimination, African Region, Progress, Challenges

## Abstract

**Background:**

Epidemics of meningococcal disease constitute a major public health challenge in Africa, affecting mostly the 24 countries of the meningitis belt. These epidemics led to a call for a call for a safe, effective and affordable conjugate vaccine against the major serogroup responsible for recent epidemics by leaders of the region.

**Objective:**

This paper documents experiences with efforts at eliminating epidemic meningitis in the African Region.

**Method:**

The meningoccocal serogroup A conjugate vaccine was developed, licensed and offered to more than 235 million people through mass vaccination campaigns in 16 countries since 2010. Future plans include providing the vaccine to the remaining countries in the African Meningitis Belt and, to implement the vaccine into routine national infant immunization programme and to organise catch-up immunization campaigns every 5 years for unvaccinated <5 year-olds who had missed their routine vaccinations.

**Results:**

The success of the project is evidenced by the large declines in cases of group A meningococcal disease since 2010, with no cases reported in vaccinated persons across the 16 countries, reflecting the highly effective nature of the vaccine. The successful control of serogroup A meningococcal disease has highlighted the need to tackle other meningococcal serogroups through development of polyvalent conjugate vaccines with the aim of eliminating epidemics of meningococcal meningitis in the African region.

## Introduction

Meningococcal disease is a major public health challenge, with large oubreaks affecting countries of the meningitis belt, which extends from Senegal in the west to Ethiopia in the east, and with an estimated total population of 500 million individuals at risk^1^,^2^. Following the deadly meningitis epidemics of 1995-96, African Ministries of Health appealed to the World Health Organization (WHO) and its partners for a solution. For the first time, African leaders demanded a vaccine for use against the disease in Africa at an affordable price (<US$1/dose). This led to the creation of the Meningitis Vaccine Project (MVP), a partnership between WHO and Program for Appropriate Technology in Health (PATH), with a grant from the Bill & Melinda Gates Foundation. The goal was to eliminate meningitis epidemics in sub-Saharan Africa through the development, testing, licensure and widespread use of affordable conjugate vaccines^3^.To reach this goal three strategies were selected: (i) to induce herd immunity through mass vaccination campaigns in the 26 countries of the belt (2010-2016), (ii) protect birth cohorts through introducing the conjugate meningitis A vaccine (MenAfriVac^©^) in routine expanded programme on immunization (EPI) for infants (<1 year-olds), and organize catch-up campaigns for unimmunised <5 year-olds every 5 years, and (iii) to institute robust surveillance and respond rapidly to new epidemics.

Here, we summarise the progress made within each of these three strategies and discuss the remaining challenges.

## Vaccination Against Meningitis

**Figure 1:**
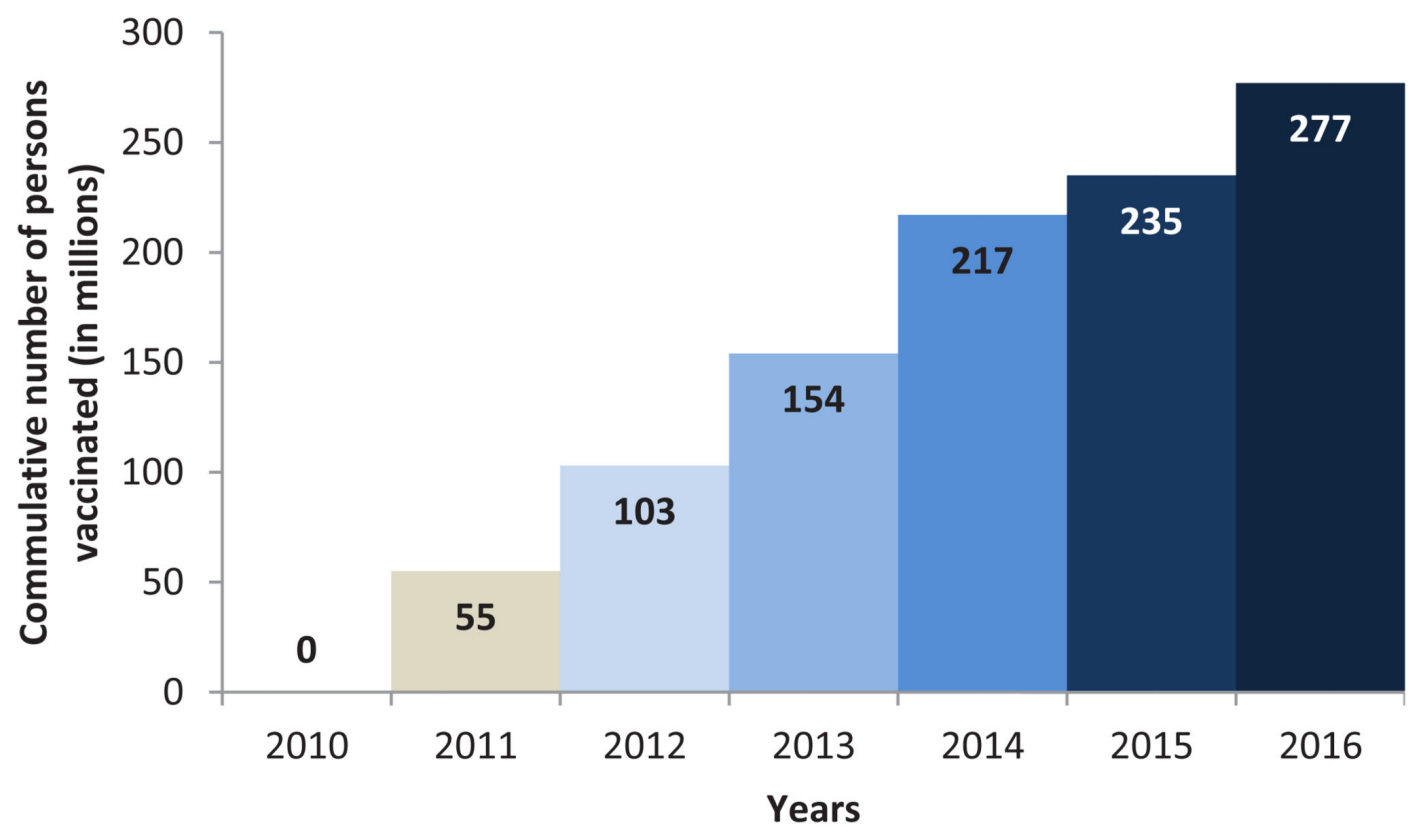
Profile of meningitis germs from 2004 to 2015 in the 16 countries that have experienced MenAfriVac© introduction

In line with this goal, a conjugate vaccine was successfully developed and deployed in the countries within the meningitis belt of Africa, through the MVP^4^–^6^. The initial strategy was to prevent outbreaks due to group A meningococci using the group A meningococcal conjugate vaccine, MenAfriVac^©^, which was developed in India, licensed by the Indian regulatory authority and prequalified by WHO. The NRAs of countries of the meningitis belt registered the vaccine prior to its introduction^7^, with detailed planning for post-marketing surveillance, including reporting, investigating and communication of adverse events following immunization. Between 2010 and November 2015, more than 235 million individuals aged 1 to 29 years were vaccinated in mass campaigns across 16 countries. An estimated 42 million more persons are planned for vaccination in the other countries of the meningitis belt. Figure 1 reflects the rapid scale-up in the number of people vaccinated with the group A meningococcal conjugate vaccine.

This large-scale vaccine introduction across the meningitis belt ountries was possible because of collaborations, partnerships, planning, training and community mobilisation of by the respective countries. This large-scale vaccination programme has been shown to be significantly cost saving for the health system and the continued use of the vaccine will ensure protection of households incomes, which in turn, will improve economic status of the people of these countries.

## Meningitis Surveillance

**Figure 2:**
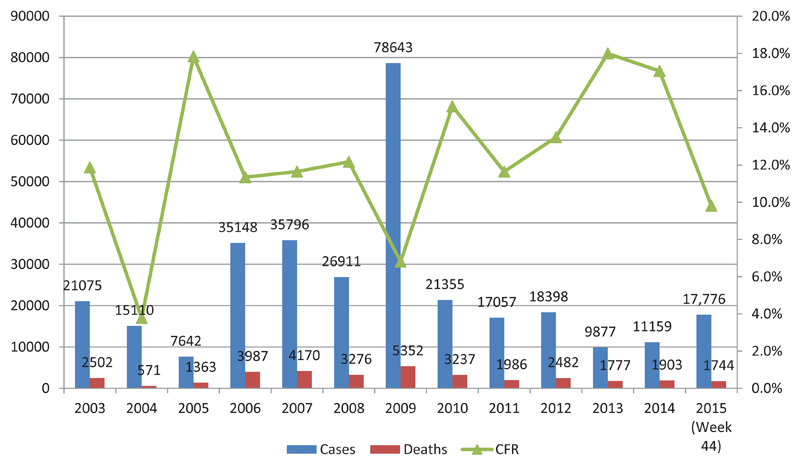
Total numbers of meningitis cases from 2004 to 2015 (confirmed) in the 16 countries that have introduced MenAfriVac©. The group A meningococcal conjugate vaccine was introduced in 2010.

**Figure 3:**
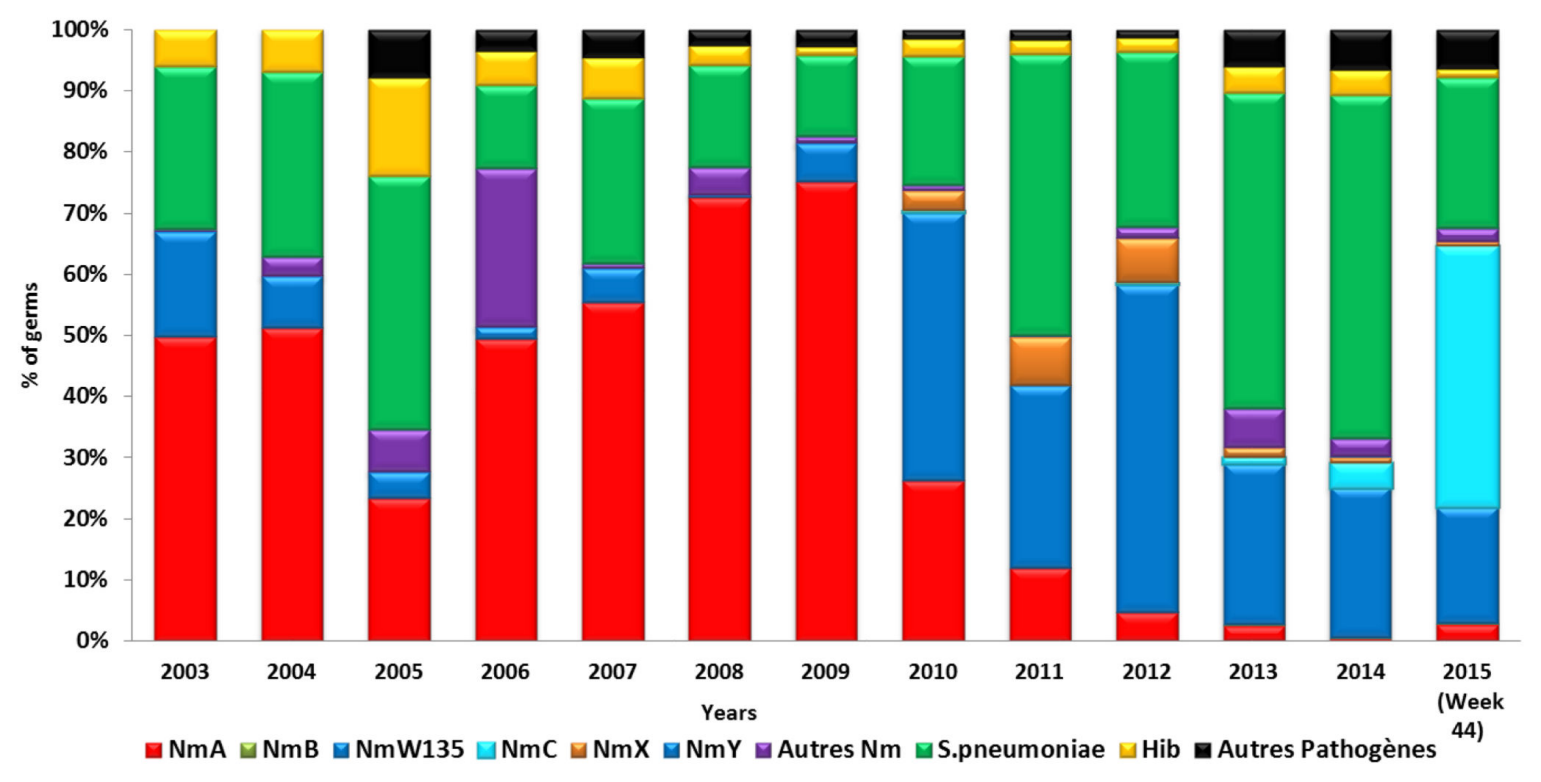
Aetiology of the pathogens responsible for confirmed meningitis cases during 2004-2015 in the 16 countries following MenAfriVac© introduction.

Enhanced surveillance for meningitis was introduced in 2003 in the meningitis belt to promptly detect, confirm and rapidly respond to emerging outbreaks. Currently, 21 are countries involved in the surveillance programme. In addition to this enhanced surveillance, a case-based surveillance programme was also introduced in 2010, just before the introduction of MenAfriVac^©^, in order to to evaluate vaccine impact. Additionally, a MenAfriNet project was introduced in 2015 to strengthen case-based surveillance in 4 countries (Mali, Niger Burkina Faso, and Togo). The vaccine has proved to be safe and effective, nd has significantly reduced the incidence and mortality due to group A meningococcal disease between 2010 and 2015^8^–^12^. Meningitis cases and deaths have remained significantly lower than the peak in 2009 (Figure 2), while the proportion of meningitis cases caused by group A meningococci has declined steadily since 2010 (Figure 3). Consequently, other causes of meningitis, such as *Streptococcus pneumoniae*, as well as serogroup W and group C *Neisseria meningitidis* have become more prominent, especially in the 16 countries that introduced MenAfriVac^©^ (Figure 3).

**Figure 4:**
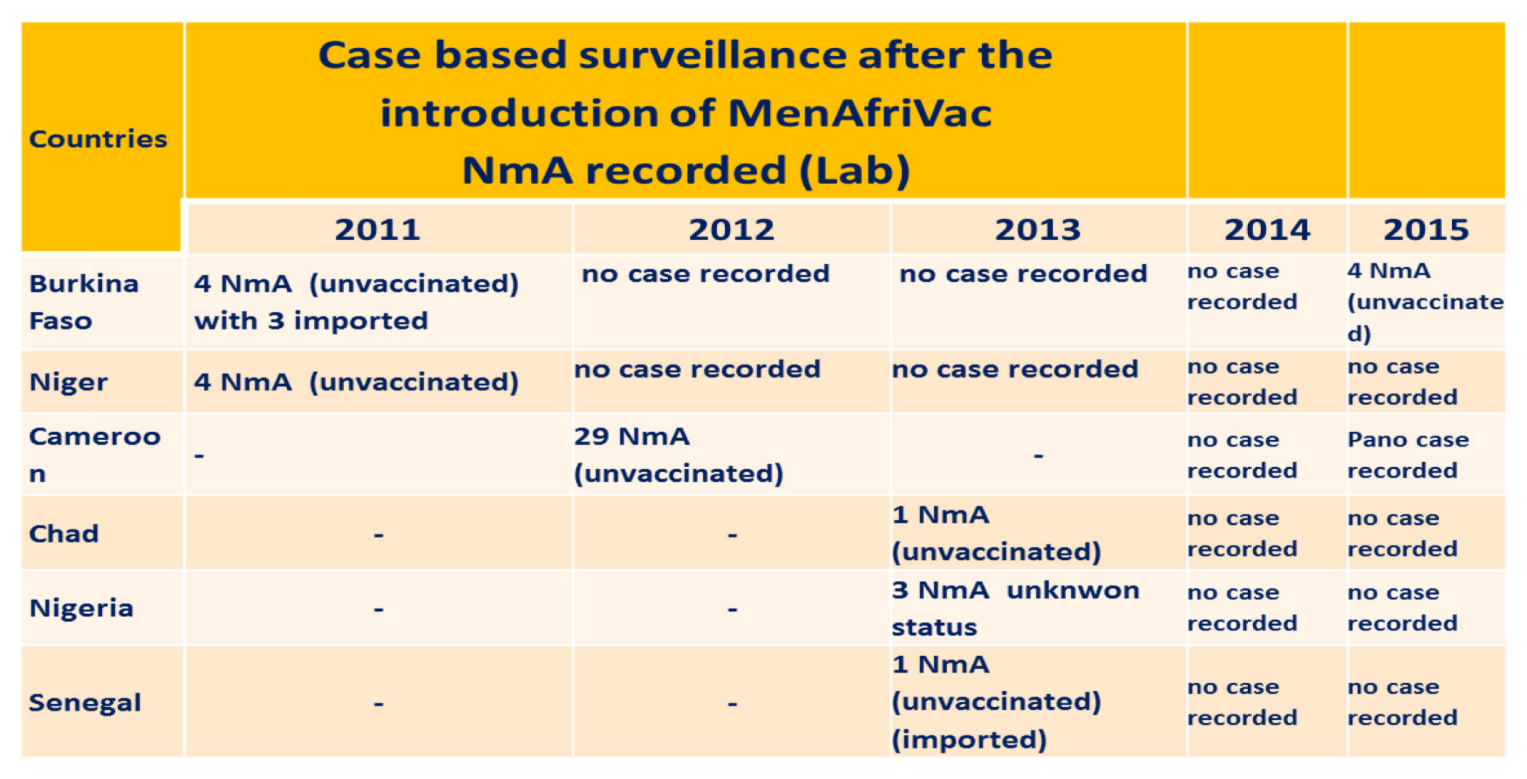
NmA confirmed reported in the countries that have implemented MenAfriVac© Campaigns

Significantly, after 2010, no case of serogroup A meningitis has reported among vaccinated people in the 16 countries, reflecting the high vaccine effectiveness of the conjugate vaccine. However, some confirmed cases due to serogroup A *Neisseria meningitidis* have been reported, all of them in unvaccinated individuals. These included 4 cases in 2015, none in 2014, 5 in 2013, 29 invaccinated cases in Cameroon in 2012, and 8 cases in 2011 (Figure 4).

## Completing Introduction and Protecting Birth Cohorts Through Routine Immunization

Despite the success of the mass immunisation programme, a lot remains to be done to ensure that epidemics of serogroup A *Neisseria meningitidis* become a disease of the past in the African region. Several activities have been planned for the coming years. MenAfriVac^©^ campaigns were implemented in some of the largest countries, the Democratic Republic of Congo, in South Sudan, and in Guinea-Bissau. Meningitis risk assessments were also conducted in Eritrea, Rwanda, Burundi and Tanzania.

The introduction of the MenAfriVac^©^ vaccine in the national infant immunisation programme and implementation of catch-up campaigns in Burkina Faso, Mali, Niger, Nigeria, Ghana, Chad, Sudan, and DRC are also planned for 2017-2019. So far, only Ghana has introduced the vaccine in routine childhood immunization programme for protecting current birth cohorts, while, for the Central Africa Republic and Uganda, routine infant immunisation and catch-up campaigns was planned for 2017.

Meningitis outbreaks in the African Belt are now due mainIy to other meningococcal serogroups, including C, X, W & Y^13^–^15^. To completely eliminate epidemics of meningococcal meningitis, an affordable and effective polyvalent conjugate vaccine will be required. Such a vaccines under development and its introduction will hopefully eliminate epidemics sof meningococcal meningitis in the African region.

In conclusion, the MVP is the first Initiative in response to a major public health problem to be addressed through a comprehensive international vaccine development and deployment strategy and exemplifies both North-south and the South-South partnerships. The successful introduction of MenAfriVac^©^ with more than 235 million people vaccinated in 16 countries is a formidable achievement. Since 2010, no case of serogroup A meningococcal disease has been confirmed among individuals vaccinated with MenAfriVac, confirming the high effectiveness predicted for this conjugate vaccine. As a consequence of this mass immunisation programme, the aetiology of meningitis in the African Meningitis Belt has changed, such that other pathogens have become relatively more prevalent causes, including Streptococcus pneumoniae and other meningococcal serogroups such as C, W and Y. Further work remains to be done; in particular, vaccination campaigns in the remaining countries in the Meningitis Belt is a priority. At the same time, countries that have already implemented mass immunisation programmes need to now focus on the introducing MenAfriVac^©^ into then routine infant immunisation programmes and offer regular catch-up campaigns to ensure high levels of indirect (herd) protection across the population. The development and introduction of a polyvalent vaccine that includes serodroups A, C, X, W, & Y, is an important requirement for the complete elimination of meningococcal disease in the African Meningitis Belt.
